# Neurological and Systemic Manifestations of Severe Scorpion Envenomation

**DOI:** 10.7759/cureus.14715

**Published:** 2021-04-27

**Authors:** Daniel A Godoy, Rafael Badenes, Sepehr Seifi, Shanay Salehi, Ali Seifi

**Affiliations:** 1 Neurointensive Care Unit/Neuro Critical Care, Hospital Carlos Malbran, Sanatorio Pasteur, Catamarca, ARG; 2 Department of Anesthesiology and Surgery, University Clinic Hospital, Valencia, ESP; 3 College of Veterinary Medicine and Biomedical Sciences, Texas A&M University, College Station, USA; 4 School of Osteopathic Medicine, University of the Incarnate Word, San Antonio, USA; 5 Department of Neurosurgery, University of Texas Health Science Center at San Antonio, San Antonio, USA

**Keywords:** systemic manifestations, scorpions, scorpion venoms, scorpion stings, neurologic manifestations, stroke

## Abstract

Scorpion envenomation is a life-threatening toxicological emergency and considered as a major public health problem, especially in endemic regions (India, Africa, Latin America); it is generally characterized by low resources and tropical or subtropical weather. Scorpion envenomation is especially fatal in the first hours, usually due to respiratory and/or cardiovascular collapse. The neurologic manifestations, triggered by multiple neurotoxic mechanisms, are varied and complex and mostly reported in children. The aim of this review is to clarify the epidemiologic characteristics and clinical manifestations as well as diagnosis and management of neurologic complications following scorpion envenomation. The management of patients with severe clinical forms is based on early recognition of the sting, antivenom serum administration, and cardiorespiratory and systemic support.

## Introduction and background

Scorpion envenomation is a life-threatening and endemic toxicological emergency. It is prevalent in certain tropical and subtropical regions of the world with low-to-moderate resources, such as Africa, India, and Latin America [1,2]. There are several species of scorpions, some with potentially lethal venom to humans [3-6]. The toxin released by the sting stimulates the autonomic nervous system (ANS), sympathetic and parasympathetic, which activates the coagulation cascade. Scorpion envenomation can produce a variety of clinical presentations, including cardiovascular (myocarditis, cardiogenic and/or distributive shock), respiratory (acute respiratory distress syndrome [ARDS]), hematological (disseminated intravascular coagulation [DIC]), renal (acute kidney injury), and neurological (seizures, autonomic dysfunction, and ischemic or hemorrhagic stroke) [4-6]. The aim of this review is to present the epidemiological characteristics and clinical manifestations as well as diagnosis and management of neurological complications following scorpion envenomation.

## Review

Epidemiology

Scorpionism is a growing public health problem; however, relevant population studies are scarce. This growth could be linked to greater awareness and registration by public health authorities or to certain changes in the environment. Phenomena such as climate change, global warming, and massive migration of the population to urban areas are factors that can contribute substantially, especially since scorpions are sedentary, nocturnal, with great adaptive capacity, and prevalent in tropical and subtropical geographical areas. More than a million scorpion stings are reported throughout the world annually, with an estimated prevalence of 20 per 100,000 inhabitants. Approximately, 5% of reported cases are severe and only 0.3% of severe cases are fatal. Scorpion envenomation may affect all individuals, but it is more prevalent in young people; 60% to 80% of them are older than 15 years but it is more severe in children principally due to minor corporal surface [1-4]. Domestic accidents in rural areas are considered the most common cause, with 80% of all recorded cases [1,2,4-6]. The incidence of stings is higher in warmer months, but it is also reported when the temperature is below 20°C. The activity of scorpions also increases during rainy seasons, especially when rain accumulation exceeds 30 mm per month [2].

Pathophysiology

The Scorpions

**Figure 1:**
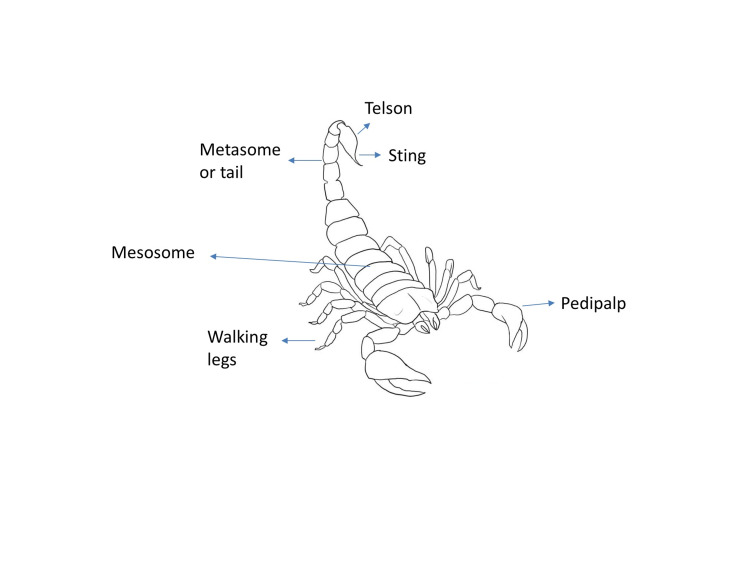
Scorpion structure

Scorpions have lived for more than 300 million years, a testament to their adaptive capacity. There are more than 1,500 species belonging to 18 families, 11 of which release potentially fatal venom (Table 1). Scorpions of the genus *Tityus* (Latin America) are arachnids (i.e., with four pairs of legs), are 5-6 cm in length, have varied colors (brown, yellow, and black), and are nocturnal habits. They have a cephalothorax and a tail, both 2.5 cm long, a clamp, and a stinger (Figure 1). Their habitats can vary from living underground, in the sand, between stones, or in sewage systems. They can survive for a long time without eating and drinking and stay underwater for up to 3 hours [4,6,7].

**Table 1:** Genus and regions of more prevalent scorpion species

Genus	Region
*Andractonus and Buthus*	North Africa
*Leirus*	Middle East
*Tityus*	South America
*Centruroides*	Central and North America
*Mesobuthus*	Asia (India)
*Parabuthus*	South Africa

Characteristics of the Toxins

The venom of the scorpion is produced in glands located in the distal portion of the tail (telson). It is composed of water-soluble polypeptides of various low molecular weights that have enzymatic and pro-inflammatory properties. The toxin stimulates the inflammatory phenomenon through the cascade stimulation of the release of cytokines, which will also regulate and amplify the immune response. Two groups of these substances stand out, those that trigger and sustain inflammation such as interleukin (IL)-1β, IL-6, IL-8, and tumor necrosis factor (TNF)-alpha, while others oppose, counterbalancing the action of the previous ones such as IL-4 and IL-10. The balance of pro- and anti-inflammatory responses will depend on the clinical manifestations of the poisoning [4,6-9].

Toxins affect various voltage-gated ion channels, principally sodium, potassium, chloride, and calcium. By acting on sodium (excitation) and potassium (blocking) channels, the toxin prolongs depolarization, mainly at the level of postsynaptic postganglionic nerve terminals of the ANS. This may cause a massive release of sympathetic (catecholamine) and parasympathetic (acetylcholine) mediators, resulting in a mixed neuroexcitatory syndrome. Experimental studies have shown that the toxin acts on brain synaptosomes, decreasing the production of GABA (gamma-aminobutyric acid), and on nerve terminals, favoring the release of excitatory and inhibitory neurotransmitters from the neuromuscular junction that would explain the clinical manifestations derived from said location [10].

**Figure 2:**
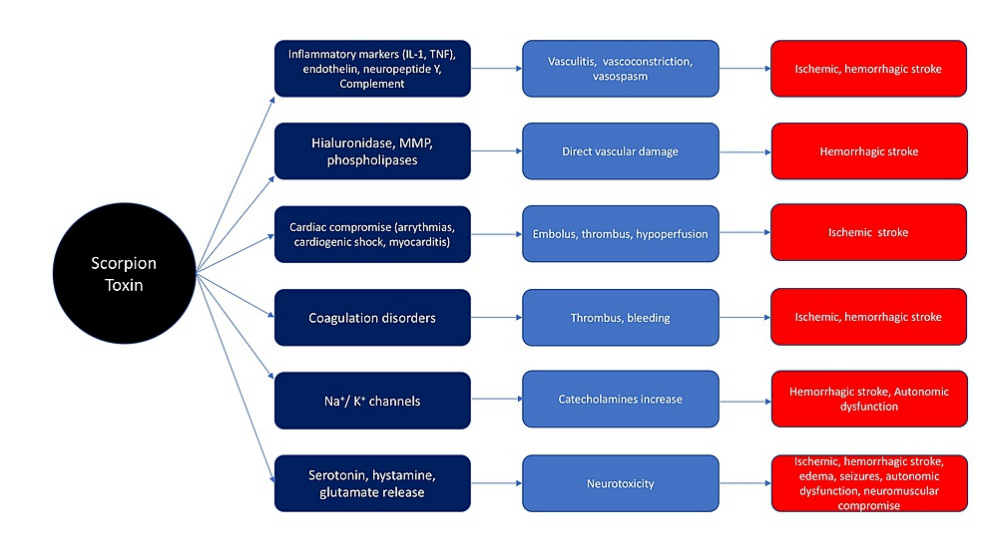
Mediators released by scorpion toxin. IL-1, interleukin-1; TNF, tumor necrosis factor; MMP, matrix metalloproteinases; Na+, sodium; K+, potassium

Additionally, the toxin stimulates the release of nitric oxide, endothelin, and neuropeptide Y. It activates the coagulation and complement systems, resulting in a vicious circle of inflammation, micro-thrombosis, and neuronal damage [4,6-9] (Figure 2).

Clinical spectrum

**Figure 3:**
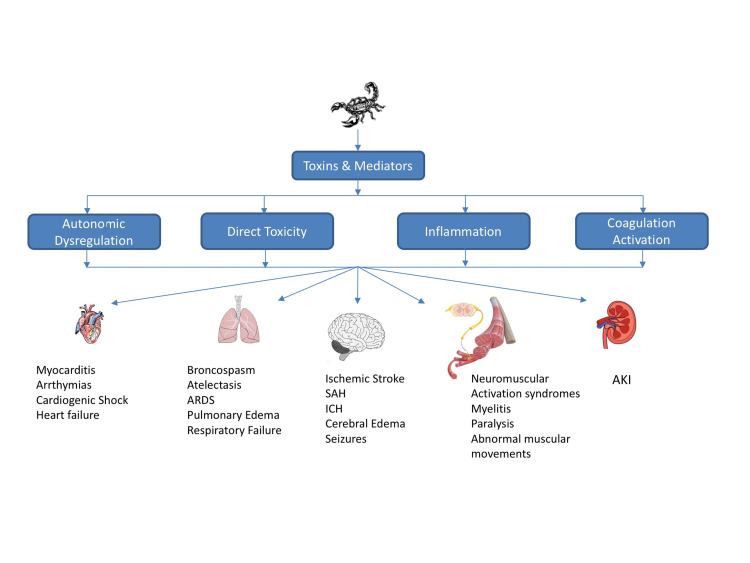
Clinical spectrum of scorpion envenomation ARDS, acute respiratory distress syndrome; SAH, subarachnoid hemorrhage; ICH, intracerebral hemorrhage; AKI, acute kidney injury

The clinical manifestations are variable with a broad spectrum, depending on the scorpion species and the plasma concentration of the toxin (Figure 3). In general, all species produce cardiovascular toxicity [4-6,8] whereas some species, such as *Centruroides* and *Parabuthus*, predominantly affect the neuromuscular system. Others, such as *Hemiscorpius lepturus*, present a syndrome characterized by late necrosis of the sting site, hemolysis, hemoglobinuria, and acute kidney injury [5].

According to the severity of the signs and symptoms, the following three stages have been established for prognostic and therapeutic purposes [2,4,6]:

● Stage I (mild): Agitation, tachycardia, pain, and erythema at the site of the sting.

● Stage II (moderate): Sweating, nausea, vomiting, tachypnea, and hypertension in addition to the above symptoms.

● Stage III (severe): Severe systemic and multi-organ life-threatening involvement.

The literature is dominated by case reports and small case series. A retrospective study on 685 children with scorpion envenomation from Tunisia showed that 81.5% of the cases corresponded to stage III. Neurological manifestations were seen in 84.7% of the patients, pulmonary edema in 63.4%, and cardiogenic shock in 14.3% [8].

Cardiovascular Manifestations

Pathophysiologically, the cardiovascular clinical picture depends on the conjunction of three mechanisms: (1) autonomic storm (adrenergic or cholinergic), (2) inflammation, and (3) direct myocardial and endothelial toxicity. Tachycardia and hypertension increase cardiac work, which, in turn, lead to myocardial dysfunction either due to an imbalance between oxygen consumption and demand or due to the presence of factors, such as vasoconstriction or spasm of the coronary arteries, that predispose to myocardial ischemia. Concomitantly, the aforementioned mechanisms favor the appearance of electrocardiographic changes and arrhythmias of various kinds. The different mediators released as a result of the action of the scorpion toxin contribute to myocarditis or stress-induced cardiomyopathy. The vascular endothelium also suffers from envenomation, ranging from mild dysfunction, moderate inflammation (vasculitis), to thrombosis. Cardiac and endothelial dysfunction can cause pulmonary edema or cardiogenic shock, and the resulting hypoxia and hypoperfusion may result in multi-organ failure [4-6,11-13].

Neurological Compromise

A. Central nervous system compromise

Mechanisms that may lead to neurological manifestations include embolic stroke secondary to myocarditis and arrhythmia, acute increase in blood pressure following autonomic storm, cerebral vessel vasospasm due to excessive catecholamine, and toxin-mediated endothelial injury and vasculitis [14-21].

Given that the toxin may provoke thrombosis and DIC, local or systemic, as pictured in figure 2, these phenomena could contribute to being one of the main causes for embolic strokes, intracerebral hemorrhage, and dural venous sinus thrombosis [14-21].

A specific family of scorpions classified as *Mesobuthus tamulus* (Indian red scorpion) has stings that reported symptoms such as ophthalmoplegia, ptosis, and respiratory failure in most patients as a consequence of neuromuscular dysfunction [10,22] and hemorrhagic or ischemic strokes in rare cases (2.6%-4%) [23,24]. Other families of scorpions such as *Tityus serrulatus* have also been reported to cause stroke [25].

Initially, patients may present no neurological symptoms [26-29]. However, persistently depressed sensorium is reported as the most common neurological symptom. Additionally, generalized tonic-clonic seizures, motor weakness, hemiplegia, dizziness, visual field defect, blindness, and dysarthria are all possible symptoms of stroke [30,31]. Seizures and coma have been signaled in the context of severe cases [11,32,33]. Cytotoxic edema has also been described [3,34].

Stroke is reported as the most prevalent cause in the adult population [1,14], with two-thirds of patients having an ischemic stroke and remaining intracranial hemorrhage [14]. Cerebral infarcts following scorpion envenomation were reported significantly in parieto-occipital, cerebellar, and capsule-ganglionic regions as bilateral and multiple defects. Bilateral anterior cerebral artery, middle cerebral artery, and posterior cerebral artery (PCA) may be involved; however, bilateral PCA is the most commonly involved artery [17,35-38]. The rare neurological presentations are thalamic and brainstem infarcts and lateral medullary syndrome [27,35,37]. Compared to infarcts, hemorrhages are less common and tend to be unilateral and single. Within the hemorrhage group, the spectrum is broad, including putaminal, lobar, intraventricular, and subarachnoid hemorrhages [18,26]. They mostly involve capsuloganglionic, cortical, and cerebellar regions. The coexistence of infarct and hemorrhage was seen in 6% of cases [25,28-30].

The mortality rate of cerebrovascular compromise following scorpion envenomation is reported at 28%; however, most of these patients suffer from other venom-mediated toxicities, including shock, coagulopathy, rhabdomyolysis, and acute renal failure, simultaneously [5,6].

Central nervous system involvement is uncommon in adults, but it is more prevalent in children [12]. In a retrospective study on complications of scorpion envenomation in children, neurological manifestations were found in 85% of cases, with irritability being the main manifestation (83.4%) followed by sweating, hyperthermia, and priapism (81.5%, 33.6%, and 48.2% of males, respectively). Furthermore, seizure and coma were found in 14.7% and 11% of children, respectively [12].

The association between age and severity of neurological compromise can be explained in part by the fact that for the same quantity of venom inoculated, the serum levels of venom will be higher in children than in adults, principally due to less corporal surface.

Moreover, the pharmacokinetics and pharmacodynamic properties of the toxin made it possible that children will have a higher uptake of the poison in the heart and the brain. Other possible but non-demonstrated explanations are that in children, the inflammatory response and catecholamines release phenomena are more intense.

B. Autonomic alterations

Cholinergic symptoms from parasympathetic activation, including nausea, vomiting, diarrhea, sweating, salivation, lacrimation, and increase in respiratory secretions, in addition to bradycardia and arterial hypotension, often predominate initially. In severe cases, an increase in sympathetic activity, such as hypertension, arrhythmias, and heart failure, becomes predominant [4-6].

C. Peripheral nervous system compromise

Activation of the peripheral nervous system causes different neuromuscular syndromes. Abnormal movements or paralysis at different levels have been described [10,22]. Guillain-Barre-like syndrome, myelitis, abnormal eye movements, facial and eye paralysis, fasciculations, and muscle jerks simulating seizures have been reported. Neuromuscular activation syndromes contribute to respiratory failure [4-6].

Diagnosis

There are no specific diagnostic tests for scorpion envenomation [4-6]. Therefore, it is important to ask questions about the event itself, investigate the different epidemiological aspects, and identify dangerous species. Table 2 depicts some certain characteristics of poisonous scorpions. In moderate-to-severe cases, cell blood count, troponin, creatinine, acid-base status, and hepatopancreatic function should be monitored.

**Table 2:** Characteristics for differentiation of venomous scorpion species

	Venomous	Nonvenomous
Sting	Unique in crescent moon form	Double
Pedipalp	Slim and elongated	Thick and short
Metasome segment	Oval	Squares
Mesosome	Usually three dark stripes	Different shades

Imaging should include chest X-ray, serial echocardiography, and brain computed tomography (CT) scan, or magnetic resonance imaging (MRI) when the clinical picture indicates neurological compromise. Although both brain CT scans and MRIs have been used for confirmation of cerebrovascular injury (CVI) in 75% and 53% of patients, respectively, MRI with diffusion restriction is preferred in differentiating dilated venules and artifacts [28].

Prognosis

In general, the prognosis is good in stages I and II [4]. In severe cases, the prognosis depends on the age, body surface, type, and size of the scorpion, as well as the time elapsed between the sting and medical assistance [4].

Management

General Principles

The first measure is to thoroughly wash the area of the sting in order to avoid dissemination of the venom to the intravascular stream. Local ice or pressure dressing can be applied. If possible, capture the specimen for analysis.

Symptomatic treatment is aimed at relieving pain by using standard analgesics and anti-inflammatory drugs. The application of warm compresses on the bite helps mitigate the pain. The routine use of antihistamines, corticosteroids, and antibiotics are not recommended unless they are administered to decrease the severity of a possible allergic reaction to the antivenom [2,4-6]. Immunization against tetanus is recommended. Antiemetics (metoclopramide, ondansetron) are necessary in cases of severe and repeated vomiting.

Intensive Care Support

The pillars on which the specific therapeutic approach is based are early recognition of the sting, specific antivenom administration, and cardiorespiratory and systemic support [2,4-6].

The first step in therapy is to assess and achieve cardiorespiratory stability. Different hemodynamic patterns may be present. In cases of distributive shock, the appropriate use of isotonic fluids (0.9% saline) is necessary to correct the hypovolemic state and hypoperfusion due to intense vasodilation. If despite the volume expansion, the mean arterial pressure is not restored, the use of vasopressors (norepinephrine) is mandatory. In cases of cardiogenic shock (heart failure, myocarditis, neurogenic cardiomyopathy), measures to mitigate pulmonary edema and low cardiac output should be used, including diuretics, inotropic drugs (dobutamine, milrinone), and in certain cases alpha and beta-blockers. The latter together with vasodilators are essential to correct and stabilize acute increase of high blood pressure [2,4-6].

Respiratory failure runs a wide spectrum. Oxygen therapy is essential. Depending on the severity of the gas exchange compromise and ventilatory mechanics, in mild and moderate, cases conventional oxygen therapy methods (nostrils, reservoir masks) can be used in order to correct hypoxemia [2,4-6]. Sometimes this is not enough, in which case the use of high-flow nasal cannulas or the initiation of non-invasive mechanical ventilation techniques (pressure support, BiPAP [bilevel positive airway pressure], CPAP [continuous positive airway pressure]) is necessary [2,4-6]. When ARDS is present, deep sedoanalgesia and protective ventilation are necessary. In general, it seems prudent to start mechanical ventilation with a controlled mode, tidal volumes between 6 and 8 mL/kg, minimum respiratory rates to ensure adequate minute ventilation (with a target PaCO_2_ of 30-32 mmHg), and FiO_2_ and PEEP (positive end-expiratory pressure) necessary to achieve PaO_2_ > 70 mmHg and/or SaO_2_ > 92%. To prevent volutrauma, plateau pressure and driving pressure should be kept < 24 and <13 cmH_2_O, respectively [6].

In severe cases, prone position and neuromuscular blockers (vecuronium, atracurium) can be employed.

Other measures inherent to the general care of the critically ill patient should be considered such as regular oral hygiene, the early use of enteral nutrition, protection of digestive bleeding, prokinetics, prophylaxis of deep vein thrombosis, eye and skincare, and early mobilization. Establish a strict program to avoid infections associated with catheters and pneumonia associated with mechanical ventilation.

At least to our knowledge, there are no guidelines or literature aimed at the specific therapy of neurological involvement secondary to scorpionism. In accordance with this and the wide range of clinical manifestations, we think that it is prudent that therapeutics should focus on the problem. In the presence of myoclonus, benzodiazepines (clonazepam) can be used. Seizure syndromes should be managed with classic antiepileptic drugs following the usual protocols for other pathologies. In cases of hemorrhagic stroke, general care measures and surgical indications are the same as other causes of intraparenchymal or subarachnoid bleeding. The ischemic stroke should be exhaustively evaluated regarding its etiology (embolic, thrombotic), and the use of thrombolytics, antiplatelet agents, or anticoagulants should be considered.

Immunotherapy

As we have mentioned before, the severity of the picture will be intimately associated with the amount of venom inoculated and the individual’s body surface. Specific treatment is aimed at blocking the action of the venom by intravenous administration of the anti-scorpion serum. The dosage depends on the severity of the condition and the type of serum available [2,4-6]. Pharmacokinetic studies have shown that the subcutaneous absorption of the venom is rapid, reaching its nadir at 60 minutes. Its distribution through the tissues ranges from 6 to 8 hours, presenting an elimination half-life of 40 hours [4]. Therefore, the administration of antivenom within the hour markedly decreases the concentration of the venom. The time between the bite and the administration of the antivenom is critical.

The best results have been reported to be achieved by neutralizing the venom within two hours of the sting [2,4-6]. The antivenom only neutralizes or blocks the circulating toxin and the one that is being absorbed in the site of the sting, not the one that is linked to the nerve terminals, nor does it neutralize the released mediator. Therefore, its use is controversial, especially in cases with severe systemic compromise [2,4-6].

Clinical Evidence

The effectiveness of antivenom has not been convincingly demonstrated. To date, there is no validated consensus, which might be due to the different reported species causing the envenomation, the small sample size, and the variation in the antivenom dosage [4-6].

A randomized controlled study on 835 patients over 10 years of age did not show any benefit; however, it only considered mild (stage I, 82.4%) and moderate (stage II, 17.6%) cases [39]. A small American study using antivenom directed against *Centruroides* species showed resolution of the clinical picture if administered within 4 hours of the bite [40]. Two randomized studies from India on both adults [41] and children [42] using specific serum against *Mesobuthus* species (red scorpion) plus prazosin have shown similar results, reporting greater efficacy with the combination compared to the use of prazosin alone.

Current evidence indicates that although serotherapy reduces the concentration of the toxin, it does not improve the clinical result [43]. However, two recent meta-analyses showed with an acceptable level of evidence that the use of serum specifically directed against *Centruroides* species is effective [44,45], although these findings cannot be extrapolated to other scorpion species.

How and How Much Serum to Administer?

The antivenom dose depends on the severity of the clinical picture and the amount of inoculated venom (generally unknown). If we take into account 30 to 50 LD50 of venom, based on the type of antivenom, generally 5 mL in mild to 20 mL or more in severe cases is required [9].

In South Africa, the antivenom is a refined equine anti-scorpion serum globulin supplied in 5-mL ampules. The standard dose is one to two ampules intravenously for both adults and children. An additional dose of 5 mL may be administered if the clinical response is considered inadequate. In the United States, immunoglobulin fractions, such as F(ab’)2, directed against Centruroides species were employed, but the efficacy of one or three doses was similar [46].

The quantity of ampules to be supplied varies due to differences in their form of production and presentation [4]. In Argentina, antivenom developed from the venom of species of the genus *Tityus trivittatus* is used, recommending the sufficient amount of antivenom capable of neutralizing an LD50 of 150 in moderate cases and 300 in severe cases. However, it is generally not indicated in mild cases [7]. In moderate cases or stage II, that is, without organic dysfunction, one to two ampules are recommended if it is manufactured at the Malbrán Institute in Buenos Aires, Argentina. If the available serum comes from Brazil (Butantan Institute), its potency is less; thus, it is suggested to use two to four ampules [7]. The Brazilian antivenom is obtained from the venom of other scorpion species, such as *Tityus serrulatus* and *Tityus bahiensis* [4]. In severe scorpionism, two to four ampules are administered if it is from Malbrán Institute and 5 to 10 ampules if it is from Butantan Institute [7].

Recommendations

Below we list certain recommendations to avoid poisoning:

1. Look for the presence of scorpions and, if possible, collect and analyze them for potentially poisonous species. It is important to evaluate the type of scorpion in order to detect poisonous species. This information is essential to establish prognosis and therapy (immunotherapy). Scorpions in general are not difficult to capture and can be tested dead or alive. Protection of hands, arms, and feet is recommended (resistant gloves, suitable shoes).

2. Periodically fumigate places prone to housing them, especially grates and drains. The creolin drives them away.

3. Establish a plan of education and communication with the population about the different types of species and habits of the scorpion, especially recognition of poisonous species.

4. Keep the surroundings of the house clean.

5. Do not go barefoot especially at night.

6. Encourage or at least not eliminate those predatory species of scorpions such as owls, meerkats, mongooses, centipedes, shrews, and tarantulas.

## Conclusions

Although scorpion venom is able to cause neuromuscular activation syndromes and stroke, the neurological complications induced by scorpion envenomation are uncommon, especially in adults. The scorpion venom is fundamentally cardiotoxic and the fatality is mostly due to cardiovascular collapse.

The severity of the toxin, delay in the presentation of the patient to a health care center, and lack of treatment policies may lead to unfavorable outcomes. Therefore, population education regarding the characteristics of species and their habitats as well as identification of dangerous species play a crucial role in prevention. Besides, further prospective studies are required to investigate the exact underlying neurological mechanisms in the context of scorpion envenomation, which, in turn, paves the way for the development of specific treatment modalities to decrease morbidity and mortality. In conclusion, early administration of specific antivenom, along with other adjuvant therapies, is critical in scorpion envenomation to reduce the associated complications such as CVI.
